# A Multilevel Investigation of Resiliency Scales for Children and Adolescents: The Relationships Between Self-Perceived Emotion Regulation, Vagally Mediated Heart Rate Variability, and Personal Factors Associated With Resilience

**DOI:** 10.3389/fpsyg.2019.00438

**Published:** 2019-03-14

**Authors:** Sjur S. Sætren, Stefan Sütterlin, Ricardo G. Lugo, Sandra Prince-Embury, Guido Makransky

**Affiliations:** ^1^Institute of Psychology, Inland Norway University of Applied Sciences, Lillehammer, Norway; ^2^Department of Psychology, University of Copenhagen, Copenhagen, Denmark; ^3^Faculty of Health and Welfare Sciences, Østfold University College, Halden, Norway; ^4^Division of Clinical Neuroscience, Oslo University Hospital, Oslo, Norway; ^5^Resiliency Institute of Allenhurst, West Allenhurst, NJ, United States

**Keywords:** heart rate variability, vagal tone, autonomic regulation, resilience, resiliency, emotion regulation

## Abstract

Personal resiliency refers to individual attributes that are related to the process of successfully adapting to the environment in the face of adverse conditions, also known as resilience. Emotion regulation is increasingly found as a core component in mental health and found to modulate individual differences in the management of emotional responses. The Resiliency Scales for Children and Adolescents (RSCA; Prince-Embury, 2006, 2007) were designed to systematically identify and quantify core personal qualities of resiliency in youth, and includes Sense of Mastery scale (MAS), Sense of Relatedness scale (REL), and Emotional Reactivity (REA) scale. The following study was first conducted to confirm the Three-Factor model of Personal Resiliency in a Norwegian student sample using factor analytic procedures. Secondly and the main purpose of the study, was to investigate if personal resiliency is associated with self-reported measures related to emotion regulation, and with resting vagally mediated heart rate variability (vmHRV) as a psychophysiological index of emotion regulation capacity. A revised scale adapted to the Norwegian sample was developed. Results indicate that protective indices related to personal resiliency are associated with both self-reported adaptive emotion regulation and outcome, and partly related to high capacity for emotion regulation indicated by vmHRV. Risk related to personal vulnerability was associated with maladaptive emotion regulation and outcome, but was not associated with emotion regulation capacity. Together the findings provide supporting evidence of both self-reported and psychophysiological correlates between emotion regulatory processes and personal resiliency indicated by RSCA.

## Introduction

The term *resilience* refers to a complex developmental process in which people do not develop mental illness despite the experience of significant threats or prolonged exposure to stressful events (Luthar et al., 2000; Masten, 2007; Kalisch et al., 2015). Previous researchers of resilience suggest that it is a positive developmental outcome that includes a dynamic interaction of the individual’s biological and psychological structure, previous and current experiences, family characteristics, and the social context (Luthar et al., 2000; Cicchetti, 2010). *Personal resiliency*, also known as *ego-resiliency*, is part of the dynamic process of resilience, and refers to personal characteristics of the individual reflecting resourcefulness and flexibility in adapting to a continuously changing environment (Luthar et al., 2000). Instead of the pathogenic approach, resilience research can be seen as a salutogenic tradition emphasizing mechanisms underlying the bioecological pattern of positive adaptation during and after a potentially traumatizing event or significant disturbances (Paton and Johnston, 2001). This includes a transaction between protective and risk factors, whereas the protective factors relative to the risk factors determine the development of adaptation or maladaptation. Whether a person develops resilience or psychopathology is therefore rooted in this complex interaction of risk and protective factors at different levels. For the last two decades, the *process of regulating emotional responses* to stressful events has been increasingly incorporated into the understanding of psychopathology development (Greenberg, 2002; Gross, 2007; Mennin et al., 2007; Aldao et al., 2010). Individual differences in *emotion regulation* and further specific regulatory strategies have both been identified as important risk factors for and protective factors against psychopathology (e.g., John and Gross, 2004; Aldao et al., 2010). It may therefore be an important characteristic of personal resiliency.

Physiological mechanisms have long been acknowledged to constitute some level of the process of resilience (e.g., Rutter, 1985; Garmezy, 1990; Masten et al., 1990; Luthar and Zigler, 1991; Luthar et al., 2000). However, it is not until recent years that a multilevel understanding, including the dynamic interaction between genes, neurobiological development and adaptation, and context at different levels (i.e., individual, family, and social), has been incorporated in resilience research (Cicchetti, 2010; Liu et al., 2017). Consequently, the current study seeks to investigate if emotion regulation at both physiological and psychological level may be related to stress adaptation and individual factors related to personal resiliency.

### The Three-Factor Model of Personal Resiliency

Based on developmental theory and research, Prince-Embury (2006, 2007, 2013, 2014) developed the *Three-Factor model of Personal Resiliency*. This model reflects aspects of an individual’s personal experience that are related to core developmental systems – *Sense of Mastery, Sense of Relatedness, and Emotional Reactivity –* and the relationship of these to one another. This is consistent with Masten’s (2007) suggestion that the human adaptive systems underlying personal resiliency is a characteristic of children and adolescent’s normal development. Sense of mastery and sense of relatedness have previously been identified as a protective personal characteristic, while the third construct emotional reactivity, is associated with risk when confronted with adversity (Prince-Embury, 2014).

*Sense of mastery* includes self-perceived competence or self-efficacy, optimism and adaptability (Prince-Embury, 2014), which build on previous definitions, theory, and research of self-efficacy and self-competence (White, 1959; Bandura, 1977, 1989a,b; Masten and Obradovic, 2006). This reflects an individual’s beliefs about his or her own capabilities to deal with the demands of prospective situations (Bandura, 1997), which both directly and through its impact on cognition, emotions, and decision-making, affects self-regulatory processes (Bandura et al., 2003). A substantial body of research suggests that individuals with greater self-efficacy, also tends to be more resilient to stress (Strecher et al., 1986; Bandura et al., 2003; Schwarzer and Warner, 2013; Bosmans et al., 2015). It is suggested that perceived self-efficacy to cope with stressors impacts the sympathetic-adrenergic reactivity, and thus buffers physiological responses (Bandura, 1982; Lazarus and Folkman, 1984; O’Leary, 1992). *Sense of relatedness* includes trust in others, access to support, social comfort, and tolerance of difference (Prince-Embury, 2014). Establishing a close and consistent relationship to a caregiver has long been recognized as a fundamental part of the process of recovering from stress (Werner and Smith, 1982; Song et al., 2013). This is not only restricted to parents but also includes other significant individuals, for example a teacher, neighbor, or peer. Different psychosocial mechanisms are suggested to explain in what way relationships increases personal resiliency (see Prince-Embury, 2014). *Emotional reactivity* represents the child’s self-perceived relative sensitivity and intensity of reaction, length of time it takes to recover after an emotional eliciting situation, and to what extent this interferes with daily functioning (Prince-Embury, 2014). Individual differences in reactions to the environment, together with mechanisms that regulates them, constitute a youth’s temperament (Rothbart, 2007). In other words, level of affectivity, intensity, and impulsivity, reflects emotional reactivity.

### Emotion Regulation

The process of regulating the generative process of emotional arousal reflects individual’s modulation of which, when, and how they experience and express their emotions (Gross, 1998, 2002). Good emotion regulation, biologically reflected by prefrontal inhibition, is furthermore crucial for adaptive functioning (for review, see Gross, 2007; Aldao et al., 2010; Thayer et al., 2012). It is considered as a key mechanism in psychological health and proposed as a transdiagnostic factor within psychopathology (Fernandez et al., 2016). The studies that directly investigate the relation between emotion regulation and adaptation in the context of adversity, demonstrate its critical importance in resilience (e.g., Cicchetti and Rogosch, 1997; Buckner et al., 2003; Cicchetti and Curtis, 2007). Two emotion regulation strategies that have been highlighted as either a protective or risk factor for an individual’s mental health is *cognitive reappraisal* and *expressive suppression* (John and Gross, 2004). Reappraisal involves an individual’s positive interpretation of a stressful situation in the attempt to reduce emotional arousal and to recover quickly after such experiences. In contrast, suppression involves an attempt to reduce emotions after the individual is already in an aroused state (Gross, 1998, 2002). Reappraisal is found related to positive cognitive, affective, and social outcomes (John and Gross, 2004), and further negatively associated with depressive and anxiety symptoms in adults and adolescents (Aldao et al., 2010; Schäfer et al., 2017). Suppression of emotions is seen as a maladaptive response found related to greater use of cognitive resources, maladaptive behavior, and a crucial component in several pathologies (Aldao et al., 2010), including depressive and anxiety in adolescents (Schäfer et al., 2017).

### Physiological Regulation and Personal Resiliency

Previous research has identified neurochemicals, neuropeptide, and hormonal mediators of stress-related responses that may be related to resilience or vulnerability (Charney, 2004; Cicchetti and Rogosch, 2007, 2012; Franklin et al., 2012). Possible neural mechanisms in the regulation of reward behavior, of anxiety and fear conditioning, and the neural basis of social behavior, have also been discussed as essential in the adaptation to adversity (Charney, 2004; Franklin et al., 2012). Recently, a common conceptual framework for the neurobiological research of resilience has been suggested and discussed (Kalisch et al., 2015). Common among what is being investigated in these studies are mechanisms related to physiological reactivity and regulation, which indeed have shown to act as powerful moderators for the variability in children’s outcome following exposure to risk and adversity (Obradović, 2012). However, the way physiological reactivity and regulation moderates resilience processes is far from fully understood. One suggestion is that both high and low sympathetic-adrenergic reactivity might be related to both adversity exposure and adaptation depending on the context (Obradović, 2012), and that regulation and following recovery via the parasympathetic nervous system might be one key mechanism that protects the individual in such experiences (e.g., Beauchaine, 2001; Porges, 2007; Hinnant and El-Sheikh, 2009; Carnevali et al., 2018). On that account, individual differences in the regulation of physiological responses to stressors might be an objective indicator of an individual’s ability to cope and adapt to adversity (Carnevali et al., 2018). A biological marker of physiological regulation related to emotional reactions may therefore contribute to our understanding of personal resiliency.

### Heart Rate Variability – A Biological Correlate of Emotion Regulation Capacity

Resting vagally mediated heart rate variability (vmHRV) is considered as a biomarker of emotion regulation capacity (Appelhans and Luecken, 2006; Thayer and Lane, 2009; Thayer et al., 2012). The sympathoexcitatory threat circuits that are active under the condition of emotional reactivity, including neural pathways between limbic structures, autonomic-, and endocrine systems, are regulated through tonic inhibitory control by the prefrontal cortex (PFC; Thayer, 2006; Thayer and Lane, 2009). The neurovisceral integration model (Thayer and Lane, 2000) states that higher prefrontal cortical activation, part of the central autonomic network, is associated with greater capacity to inhibit maladaptive behavioral, emotional, and physiological responses to emotionally demanding situations. PFC regulates autonomic responses via vagus-mediated influence on the parasympathetic nervous system (Thayer and Lane, 2000; Thayer et al., 2012), hence adapting the heart rate to the internal and/or the external environment. Greater variety between consecutive heartbeats (inter-beat intervals) reflects greater parasympathetic input via vagally mediated and prefrontal cortical modulated influences, resulting in higher resting vmHRV. In accordance to the *Polyvagal Theory* (Porges, 2007), parasympathetic influence on the heart rate operates as a ‘brake’ to give flexibility of the physiological system to regulate sympathetic activation. This psychophysiological process includes inhibiting sympathetic arousal when the environment is interpreted as “safe,” but also disinhibiting the sympathetic-adrenergic system and mobilizes coping behavior under the condition of perceived threat. Together it facilitates flexible adjustment to environmental demands. The theory also states that social affiliative behavior is part of the same adaptive emotion regulatory system, and furthermore, that high vmHRV, social competence, and active engagement with the environment are interrelated processes (Porges, 2007). vmHRV is, across age groups, supported as a valid biomarker for top-down regulation of emotional, cognitive, and behavioral processes (Holzman and Bridgett, 2017). Specifically, vmHRV is an objective index of emotional capacity to adapt and recover after experiencing significant stress (Thayer and Lane, 2000; Appelhans and Luecken, 2006; Segerstrom and Nes, 2007; Hastings et al., 2008; Thayer et al., 2012). Growing evidence also supports the relation between self-reported emotion regulation and vmHRV, in both adolescents and adults (Fabes and Eisenberg, 1997; Vasilev et al., 2009; Williams et al., 2015; Visted et al., 2017). Recently, vmHRV has been proposed as a physiological marker of stress resilience (Carnevali et al., 2018). It may therefore serve as a valuable and objective index of personal resiliency, in supplement to self-report measures.

To date, empirical work investigating vmHRV and self-reported personal resiliency directly is scarce, but available studies point to associations to both protection and greater risk for developing psychopathology (e.g., Souza et al., 2013; Chalmers et al., 2014). In what way vmHRV relates to resiliency needs further thorough investigation. The vagal tank theory (Laborde et al., 2018), postulates three levels of adaptability (i.e., resting, reactivity, and recovery) to illustrate the constant role of cardiac vagal control on self-regulation before, during and after, an individual copes with a significant emotional demand. Each of these levels provides possible predictions in how vmHRV may be related to resiliency (Laborde et al., 2018). The target of this study is to investigate if *resting* cardiac vagal control (i.e., resting vmHRV) in adolescents is associated with an adaptive resiliency profile. Another important aspect is that resiliency as a multisystem phenomenon influenced by several biopsychosocial factors (e.g., genetics, stress hormones, neurotransmitter systems, family, friends, school, society; Cicchetti, 2010). This also implies that vmHRV may relate to the individual’s resiliency differently. For example, a child developed in a resource-facilitating environment (i.e., promoting mastery and social support), but with low vmHRV, can still have high resiliency. In contrast, an individual without such environment such an environment can still have high resiliency because of other personal resources, such as high vmHRV.

According to HRV theory and previous empirical work, higher resting vmHRV predicts higher emotion regulation and stress management, higher executive performance and better social functioning (Thayer and Lane, 2000; Porges, 2007; Staton et al., 2009; Thayer et al., 2012; Laborde et al., 2018). In fact, replicated findings do suggest that young adults who cope adaptive to severe and acute stress, both score higher on self-reported resilience measures and display higher levels of resting vmHRV (Souza et al., 2007, 2013). Higher resting vmHRV in both children and adolescents has been shown to be positively associated with resiliency-related behavior, such as better social functioning and increased use of external emotion regulation such as social support seeking (Eisenberg et al., 1995; Kok and Fredrickson, 2010; Geisler et al., 2013; De Witte et al., 2016). In addition, high resting vmHRV can also buffer adolescents *against* adverse family adversities (Katz and Gottman, 1995, 1997; Blandon et al., 2008). Together, this may suggest higher resting vmHRV as one of several important protective psychophysiological mechanisms underpinning personal resiliency. In contrast, low resting vmHRV has been found related to risk for development of psychopathology (Chalmers et al., 2014; Gillie and Thayer, 2014; Sgoifo et al., 2015). Parental reports of adolescents’ impulse control deficits are also negatively associated with vmHRV (Allen et al., 2000). This is additionally supported by the fact that vmHRV has been found related to both cognitive and autonomic responses to emotional reactions in children and adolescents (Chapman et al., 2010), which furthermore supports a unified psychophysiological regulatory system. In other words, lower resting vmHRV may be related to maladaptive emotional responses and additional risk after being exposed to significant stress.

## Aim

The aims of the following study are twofold. First confirm Prince-Embury’s Three-Factor model of Personal Resiliency in a Norwegian student sample using factor analytic procedure, and secondly, to investigate associations between components of personal resiliency and emotion regulatory processes. Protective indices related to personal resiliency are expected to be positively associated with self-reported adaptive emotion regulation, while self-reported dysregulation of emotions is expected to be related to greater self-reported risk indicated by RSCA. In regard to psychophysiological correlates between vmHRV and RSCA, we expect a positive association between vmHRV and Sense of Mastery, and between vmHRV and Sense of Relatedness. Finally, in light of the theoretical underpinning of Emotional Reactivity (Prince-Embury, 2014), we expect a negative association between vmHRV and self-reported emotional reactivity indicated by RSCA. Combining both self-report and psychophysiological measurements makes the present study among the first to apply a multilevel approach to investigate the association between personal resiliency and emotion regulatory processes.

## Materials and Methods

### Participants

For factor analysis, 180 high school students (64% females, mean age = 17.04, *SD* = 1.35) were recruited through convenience sampling from different parts of Norway. A subgroup of this sample (*N =* 45, 69% females) born 1998/1999 (i.e., 17–19 years at data collection), were selected for additional self-reported and psychophysiological data collection. These were recruited from two high school classes and everyone had chosen psychology as an elective subject.

### Questionnaires

#### Resiliency Scales for Children and Adolescents

The RSCA was used to assess self-reported personal resiliency. The RSCA is a 64 item self-report questionnaire comprising three global scales; Sense of Mastery (MAS; 20 items), Sense of Relatedness (REL; 24 items), and Emotional Reactivity (REA; 20 items; Prince-Embury, 2006, 2007). Each global scale consists of conceptually related subscales. MAS consists of optimism, self-efficacy and adaptability. REL consists of comfort, trust, perceived support, and tolerance of differences with others. REA consists of sensitivity, recovery, and impairment in emotional reactivity. Each item is assessed using a 5-point Likert-type scale ranging from (1: never to 5: almost every time). Previous studies have reported α (Cronbach’s alpha) for the three global scales ranging from 0.90 to 0.94 for American students between ages 15 and 18 (Prince-Embury and Courville, 2008). Furthermore, confirmatory factor analysis (CFA) has shown that the scale fits the Three-Factor model in three youth samples between ages 9 and 18 from the United States (Prince-Embury and Courville, 2008).

The RSCA items were translated from English to Norwegian following the criteria set by Brislin (1970). The translators both had over 20 years’ experience each within developmental psychology and both were fluent in English and Norwegian. The original English version was translated by a native Norwegian speaker while the back translation was conducted by the English native speaker. An independent Norwegian psychologist with knowledge of resilience conducted a comparison of the original English version and back translated version. As a result, a common single version was developed that was appropriate for the Norwegian culture. An adapted version followed by the results from CFA was used to assess self-reported personal resiliency.

#### Self-Efficacy Questionnaire

A Norwegian translation of 10-item General Self-Efficacy Scale (GSE; Schwarzer and Jerusalem, 2010) was used. Its psychometric properties have been investigated repeatedly in several cultures, where coefficient alphas between 0.75 and 0.91 have been reported, reflecting high internal consistency (Scholz et al., 2002; Luszczynska et al., 2005).

#### Emotion Regulation Questionnaire

A Norwegian translation of Emotion Regulation Questionnaire was used to measure participants emotion regulation strategies (ERQ; Gross and John, 2003). The ERQ is a 10-item questionnaire that measure individual differences in the use of emotion regulation strategies: reappraisal (six items) and suppression (four items). Each item is scored on a 7-point Likert scale (strongly disagree to strongly agree). The scale has reported good reliability (Reappraisal: Cronbach’s α = 0.91; Suppression: Cronbach’s α = 0.80), and been used in studies using vmHRV (e.g., Hollenstein et al., 2012) and resilience (e.g., Waugh et al., 2008).

#### Strengths and Difficulties Questionnaire

Participants’ strengths and difficulties related to emotional dysregulation were measured by the Norwegian standardized self-report version of the Strengths and Difficulties Questionnaire for children and adolescents (SDQ; Goodman, 1997; Kornør and Heyerdahl, 2014). SDQ is a widely internationally used screening tool for assessing and monitoring psychiatric disorders in children and adolescents aged 4–17 years. It consists of five subscales; Emotional Problems scale, Conduct Problems scale, Hyperactivity scale, Peer Problems scale, and Prosocial scale. Each subscale contains five items that together make up a Total Difficulties score, with the exception of the prosocial scale. Except for the prosocial scale, higher scores indicate more problems, whereas a higher prosocial score indicates fewer difficulties in prosocial behavior. SDQ has repeatedly shown good reliability and validity, in both Norwegian and other populations (Goodman, 2001; Kornør and Heyerdahl, 2014; Bøe et al., 2016).

### Psychophysiological Assessment

Participants were asked to report their use of caffeine and tobacco, and participants using beta-blockers or other drugs that may influence cardiac arrhythmia were excluded. vmHRV was measured first by placing three IOM finger sensors on the participant’s non-dominant hand, which is a biofeedback system that measures heart rate. This is through the recording of photoplethysmography (PPG), also known as blood volume pulse (BVP), using Alive Software (Alive^TM^ by Somatic Vision, Inc.). PPG is found to be a highly useful, non-invasive, and low-cost alternative to standard physiological measures (i.e., electrocardiogram) gathering vmHRV information (Lu et al., 2008). All participants were asked to refrain from caffeine for 2 h prior to assessment and none of the participants reported that they used tobacco. The assessment was carried out in a period of 4 days during school hours, and measured under identical conditions in sound-attenuated rooms. First, rapport was established and participants were informed about vmHRV and the assessment procedure. An instruction to breathe consistent in a naturally pace throughout the procedure without moving their arm or body was then given. They were asked to not use their phone and to sit back and rest during assessment. PPG was recorded during a 7- to 8-min period where the participant was left alone in the room. PPG data used for analysis was drawn out from the first 150 s and last 50 s. If significant artifacts from hand movement or other disturbances were detected within this interval, the highest quality 5-min interval was selected.

For vmHRV analysis, the two most frequently used vmHRV indices of time- and frequency domain were used. These are the root mean square of successive differences (rMSSD) and the high frequency component (HF; Fast-Fourier-Transformation, bandwidth 0.15 to 0.40 Hz), which are suggested to be the best indices of vmHRV (Task Force of the European Society of Cardiology, 1996). Sex stratified normative data of vmHRV measured in supine rest state in healthy adolescents between 12 and 17 years with normal BMI (non-athletes), shows that for rMSSD (ms), girls have a minimum 12.80 and maximum 256.30, and boys minimum 9.10 and maximum 350.40 (Sharma et al., 2015). Another comparable study applying sitting rest condition during measurement reports range for rMSSD between 9.61 and 188.08 (De Witte et al., 2016).

### Statistical Analysis

Mplus (Muthén and Muthén, 2012) was used for performing the CFA. The items were treated as ordinal variables. The fit of a hierarchical model made up of three global factors and the 10 sub-scales based on the theoretical structure of the model was tested (Prince-Embury and Courville, 2008). Three goodness-of-fit indices according to the criteria outlined in Hu and Bentler (1999) were used to evaluate the fit of the CFA. Acceptable fit was determined based on CFI and TLI scores ≥0.90 and an RMSEA score ≤0.06.

Normality was visually inspected. Strength and Difficulties total score (i.e., SD Total), Piers Harris Behavior Adjustment score (i.e., PH BehAdj), and vmHRV index (i.e., rMSSD and HFms^2^) were transformed to achieve normality.

The associations between convergent measures and RSCA, and between vmHRV and RSCA, were investigated through Pearson correlation tests by using SPSS Software Package (version 24, IBM Corp., 2016). Literature suggests that higher vmHRV may be related to protective characteristics of personal resiliency and lower vmHRV may relate to greater risk. However, resiliency as a multisystem phenomenon implies that vmHRV may influence its related components differently, and analysis of vmHRV sub groups may therefore contribute to the explanation of how it is related to resiliency. Conducting median-split provides one statistical way to assess this complex relationship in more detail (Iacobucci et al., 2015). *Post hoc* investigation of hypotheses regarding vmHRV and RSCA were therefore conducted for high and low vmHRV separately, whereas groups were determined through median split (rMSSD median = 1.83).

Inter-beat intervals (IBIs) were detected via R-peak extraction and HRV was analyzed using ARTiiFACT software (Kaufmann et al., 2011). Detection of artifacts was done both by a distribution-based detection algorithm and an additional visual inspection. Erroneous IBIs were replaced by means of cubic spline interpolation.

### Ethics Statement

The study conformed to institutional guidelines of applying to the Norwegian Social Science Data Services (NSD) ethical guidelines for experimental studies. Formal application was not required after initial NSD online form was filled out. Only non-identifiable and non-health related data was used in this research. All participants provided written informed consent, and were debriefed about the study’s purpose after completing the data collection. In accordance with NSD guidelines, informed consent from parents was not collected when all participants were 16 years or older. Participants were informed that they could withdraw from participation at any time and without any consequences throughout and after data collection.

## Results

### Confirmatory Factor Analysis of RSCA

The results of the CFA are presented in supplementary data. The initial CFA with all 64 items resulted unacceptable fit indices: CFI = 0.824, TLI = 0.817, and RMSEA = 0.058. Furthermore, item fit statistics indicated that five items (REL: item 23, 24; REA: 18, 19, 20) did not fit the model as intended. No apparent adaptation problems were identified when comparing the English and Norwegian versions of these items. The five items were thus eliminated and a new analysis was conducted with the revised scale. The results of the fit to the revised model with 59 items resulted in acceptable fit indices: CFI = 0.908, TLI = 0.904, and RMSEA = 0.045. Furthermore, all items loaded on their intended subscale as predicted. The Cronbach’s α for the global scales were MAS = 0.875, REL = 0.903, and REA = 0.871 for the Norwegian RSCA, which is consistent with other studies (Prince-Embury and Courville, 2008; Prince-Embury, 2014). We conclude from step one that a revised version of the RSCA with 59 items fits the theoretical framework of the model, and is a valid measure of personal resilience in the sample of Norwegian students used in this study.

### Self-Reported and Psychophysiological Correlates

#### Descriptive Statistics

Table 1 provides descriptive data of all variables. Characteristics for the revised RSCA, including the three global scales MAS, REL, and REA of the RSCA, are presented in Table 1. RSCA-N scores are reported as raw scores and cannot be compared with United States standardization. Table 1 provides also characteristics of self-report measurements and vmHRV indices. The latter includes vmHRV parameters (i.e., rMSSD, HFms^2^, and pNN50) as well as other vmHRV indices (i.e., mean HR and LFabs), and were only included for the sake of comparability with other studies and replicability. These measures will not be included in further analysis.

**Table 1:** Descriptive statistics for vmHRV, Resiliency Scales for Children and Adolescents, Self-Efficacy Questionnaire, Emotion Regulation Questionnaire, and Strength and Difficulties Questionnaire.

	*N*	Minimum	Maximum	Mean	*SD*
RMSSD	41	34.62	131.33	68.71	23.6
MEANHR	41	57.07	91.2	69.38	8.94
pNN50	41	13.54	69.82	42.69	14.44
LF	41	105.8	7621.27	2169.52	1812.79
HF	41	92.94	6145.32	1889.46	1509.83
MAS	41	56	91	74.29	7.64
REL	41	68	107	88.22	8.92
REA	41	25	69	41.22	10.32
SEQ	41	1.7	3.7	3	0.42
Reappraisal	41	1.8	6.8	4.53	0.8
Suppression	41	1	6.8	3.97	1.29
Emotional Problems	37	0	9	4.24	2.39
Conduct problems	38	0	6	1.84	1.57
Hyperactivity	37	1	8	4.08	2.06
Peer problems	38	0	5	2.13	1.42
Prosocial	38	5	10	8.32	1.63
SD total	37	6	22	12.35	4.48

*rMSSD, root Mean Square Successive Difference; HF, high frequencies; LF, low frequencies; MAS, Sense of Mastery scale; REL, Sense of Relatedness scale; REA, Emotional Reactivity scale. SEQ, Self-Efficacy Questionnaire; ERQ, Emotion Regulation Questionnaire; SD total, Strength and Difficulties total score.*

Four participants were excluded because of missing participant code between questionnaires and vmHRV. N varies across self-reported measures because of invalid answering (i.e., subject did not answer the item or selected two alternatives). Results from vmHRV analysis indicate that the total sample is well within the normal range compared to other studies (Sharma et al., 2015; De Witte et al., 2016). Sex differences where non-existent [*t*(15) = 0.198, *p* = 0.844].

#### Self-Reported Correlates

Table 2 provides correlations between RSCA global scales (MAS, REL, REA), self-reported measures, and vmHRV (i.e., RMSSD). MAS correlated positively with Self-Efficacy, Reappraisal, and Prosocial behavior score. MAS did also correlate negatively with Emotional Problems and Conduct Problems together with the SDQ total score. Effect sizes may overall be interpreted as moderate to strong (Cohen, 1992). Similar to MAS, REL was also correlated with Self-Efficacy and Reappraisal, although not as strong. REL was further found negatively correlated with emotional problems, conduct problems, and SDQ total score. In contrast to MAS, REL was also found negatively correlated with peer problem score. While the association between REL and Reappraisal may be interpreted as small to moderate, the other effect sizes for REL are seen as moderate to strong (Cohen, 1992).

**Table 2:** Pearson correlations vmHRV, RSCA global scales, Self-Efficacy, Emotion Regulation, Strength and Difficulties.

		1	2	3	4
1	RMSSD	1			
2	MAS	0.126	1		
3	REL	0.204	0.548**	1	
4	REA	0.087	-0.285*	-0.414**	1
5	SEQ	0.131	0.691**	0.382**	-0.16
6	Reappraisal	-0.067	0.425**	0.264*	-0.002
7	Suppression	-0.202	-0.136	-0.149	-0.346*
8	Emotional problems	-0.226	-0.433**	-0.415**	0.197
9	Conduct problems	0.065	-0.481**	-0.462**	0.414**
10	Hyperactivity	0.155	-0.104	-0.189	0.423**
11	Peer problems	-0.313*	-0.015	-0.306*	-0.091
12	Prosocial	0.028	0.344*	0.407**	-0.268
13	SD total	-0.117	-0.442**	-0.560**	0.403**

*^∗^Correlation is significant at the 0.05 level (one-tailed). ^∗∗^Correlation is significant at the 0.01 level (one-tailed). rMSSD, root Mean Square Successive Difference; HF, high frequencies; LF, low frequencies; MAS, Sense of Mastery scale; REL, Sense of Relatedness scale; REA, Emotional Reactivity scale; SEQ, Self-Efficacy Questionnaire; ERQ, Emotion Regulation Questionnaire; SD total, Strength and Difficulties total score.*

RSCA risk index Emotional Reactivity (REA) correlated positively with conduct problems, hyperactivity, and SDQ total score. In addition, REA correlated negatively with suppression and peer-problems, although it did not reach level of significance for peer-problems (*p* = 0.052). Across associations, effect sizes are interpreted as moderate to strong (Cohen, 1992). Important to note, while adaptive emotion regulation indicated by Reappraisal score (i.e., ERQ) did correlate with MAS and REL, it did not correlate with REA. In contrast, maladaptive emotion regulation indicated by Suppression score did only correlate with Emotional Reactivity, although this relationship was found negative.

#### Psychophysiological Correlates

In line with the majority of previous studies using vmHRV, correlations indicated that HFms^2^ power and RMSSD were highly correlated (*r* = 0.890, *p* < 0.001) (Goedhart et al., 2007). In accordance with literature, only one vmHRV index is reported (RMSSD).

In Table 2, correlations between overall vmHRV (rMSSD) and RSCA scores (MAS, REL, REA) are provided. Looking at the total sample, vmHRV was not correlated with any of the RSCA global scales. However, vmHRV was found positively correlated with Trust (*r* = 0.341, *p* = 0.015), which is one of four sub scales in REL.

### *Post hoc* Analysis

Table 3 provides the results from *post hoc* analysis. Investigation of the relationships between high/low vmHRV defined by median split and RSCA global scores revealed several significant associations. In the low vmHRV group, no significant associations were identified (*p* > 0.05). In contrast, in the high vmHRV group, MAS and REL were significantly positively correlated with vmHRV. Effect sizes can be interpreted as moderate to strong (Cohen, 1992). REA was negatively correlated with high vmHRV, but did not reach level of significance.

**Table 3:** *Post hoc* correlations low vmHRV, high vmHRV, and RSCA global scales and sub scales.

	Low RMSSD (*N* = 21)	High RMSSD (*N* = 20)
MAS	-0.133	0.504*
REL	-0.01	0.399*
REA	-0.046	-0.289
Optimism	-0.143	0.294
Self-efficacy	-0.113	0.481*
Adaptability	-0.012	0.490*
Trust	0.177	0.485*
Support	0.138	0.29
Comfort	-0.058	0.452*
Tolerance	-0.047	0.192
Sensitivity	-0.271	-0.107
Recovery	0.071	-0.345
Impairment	0.171	-0.287

*^∗^Correlation is significant at 0.05 level (one-tailed). ^∗∗^ Correlation is significant at 0.01 level (one-tailed). rMSSD, root Mean Square Successive; MAS, Sense of Mastery scale; REL, Sense of Relatedness scale; REA, Emotional Reactivity scale.*

To investigate these associations in more detail, correlations for high vmHRV and RSCA subscales were conducted (Table 3). As expected, High vmHRV did correlate moderate to strong with MAS subscales self-efficacy and adaptability, but did not reach significance for optimism (*p =* 0.084). For REL subscales, high vmHRV did correlate with trust and comfort, but not significantly with support (*p* = 0.089). The correlation for trust may be interpreted as strong, while the correlation for comfort as moderate to strong. For REA subscales, there was no significant correlations, but recovery subscale did, however, indicate a tendency for a negative association (*r* = -0.345, *p* = 0.068).

## Discussion

The aim of the present study was to investigate the Resiliency Scales for Children and Adolescents (Prince-Embury, 2006, 2007) with factor analysis, and to investigate the role of self-reported emotion regulation and vmHRV as a psychophysiological index of emotional regulation capacity in personal resiliency. Together, the study investigated self-reported and psychophysiological correlates of emotion regulation to factors of personal resiliency in a Norwegian student sample. The study is among the first to apply a multilevel approach in the evaluation of personal resiliency (Cicchetti, 2010).

### Findings

Results from the initial CFA with all 64 items from the original version of RSCA indicated that the data did not fit the Three-Factor model as intended. Five items were identified as problematic. Comparison and evaluation of items revealed that cultural differences could not explain their low factor loadings. Items were therefore eliminated before a new analysis was conducted. The revised scale with 59 items resulted in an acceptable fit, and all items loaded on their intended subscale as predicted. With an acceptable model fit and good Cronbach’s alphas (ranging 0.87–0.90), the revised scale was in accordance with the theoretical framework of the Three-Factor model (Prince-Embury, 2006, 2007), and was used for further analysis.

The three global scales MAS, REL, and REA (RSCA-N), were consistently related with emotion regulatory processes and outcomes. Individual’s using cognitive reappraisal as a strategy to regulate their emotions have previously found to function well emotionally, cognitively and behaviorally (John and Gross, 2004), and further associated with good mental health (Aldao et al., 2010) and negatively associated with psychopathology in adolescents (Schäfer et al., 2017). Consistent with this, the protective indices MAS and REL were positively related to cognitive reappraisal, and further related to adaptive behavior indicated by prosocial behavior. An inverse relationship was found in regard to both emotional problems and conduct problems, which further supports the protective function of MAS and REL. In addition, peer problems were not related with MAS, but with REL. This might suggest that Sense of Relatedness uniquely captures important aspects of youths interpersonal functioning related to personal resiliency. Emotional Reactivity as a risk index in RSCA was negatively associated with maladaptive emotion regulation indicated by suppression. Suppression is previously found related to maladaptive emotional responses (John and Gross, 2004) and development of psychopathology (Aldao et al., 2010; Schäfer et al., 2017). The finding is therefore in contrast to previous literature. However, it may be that Emotional Reactivity scale captures other aspects of maladaptive responses than specific maladaptive emotion regulation processes. In support of this notion, Emotional Reactivity was not found related to emotional problems, but positively related to both conduct problems and hyperactivity.

Inconsistent with our hypotheses, overall resting vmHRV was not found to be related to any of the three RSCA global scales (MAS, REL, REA). vmHRV was, however, found to be correlated with the Trust subscale (REL). The explanation for these findings remains unclear as current results are inconsistent with both theory (Porges, 2007) and previous studies (e.g., Allen et al., 2000; Souza et al., 2007, 2013; Blandon et al., 2008; De Witte et al., 2016). It may be speculated that low emotion regulation capacity (i.e., low vmHRV) for healthy adolescents living in a socially resourceful and functioning environment, may not be a significant risk factor for either self-perceived mastery, relatedness, nor emotional reactivity. In contrast, it is suggested that high capacity for regulating emotional arousal may dispose individuals to be more socially and cognitively competent (Eisenberg et al., 1995; Staton et al., 2009) and further apply adaptive emotion regulatory processes (Blandon et al., 2008; De Witte et al., 2016). In line with this, higher vmHRV may function as a protective factor facilitating adaptation independently or in addition to, other biopsychosocial protective factors. To investigate this further, analyses for vmHRV sub groups were done by conducting a median split (Iacobucci et al., 2015). *Post hoc* investigation of adolescents with high and low vmHRV separately, indicated several interesting associations. Partly consistent with our hypotheses, higher vmHRV was associated with increased self-reported mastery and relatedness. This was not the case for emotional reactivity, although it was a clear tendency for the recovery subscale. Due to sample size reduction after conducting median-split and thereby reduced power, this must be interpreted carefully. The findings are, however, important to discuss.

For sense of mastery, the associations were especially applicable for self-efficacy and adaptability subscales. Currently, there is a lack of research investigating the association between vmHRV and sense of mastery or efficacy. However, according to Bandura (1989b), “Development of self-regulatory capabilities requires instilling a resilient sense of efficacy as well as imparting knowledge and skills” (p. 733). Consistent with this statement, our study indicated that both high vmHRV and self-reported adaptive emotion regulation (i.e., reappraisal) were related to greater sense of master and efficacy. The explanation for this relationship is, however, unclear and future work is needed to examine possible mechanisms explaining how vmHRV may be one psychophysiological component associated with sense of mastery. In line with previous research (e.g., Fabes and Eisenberg, 1997; Blandon et al., 2008; De Witte et al., 2016), children and adolescents with greater vmHRV may be more disposed to apply adaptive emotion regulation and by that, adapt successfully with the environment. This may in turn also lead to increased probability for exposure of mastery experiences, and hence, increase self-efficacy. Additionally, with the suggestion that high vmHRV, social competence and engagement with the environment, are interrelated processes (Porges, 2007), adolescents with higher vmHRV may increase their self-efficacy both directly and indirectly in a mutual supporting social environment. High vmHRV may be one of many related components to self-efficacy. Other factors such as social comparison and feedback (Bandura, 1977) may be more important for those with low vmHRV.

In line with the *Polyvagal Theory* (Porges, 2007), vagally mediated social affiliative behavior is part of the autonomic regulatory system, reflected by that high vmHRV is thought to support social engagement. This notion is supported by the findings that adolescents with higher vmHRV were associated with reporting greater self-reported sense of relatedness. The association can be explained in several ways. Individuals with high vmHRV may seek out social support because they have learned throughout their lives that it is a useful emotion regulation strategy by recovering quickly after distress. Individuals with high vmHRV may be more likely to have a good social network, or the opposite, that good social network facilitates high vmHRV. This study suggest, however, that adolescents with high vmHRV may be related to perceive their social network as an external buffer against stress, which is in line with previous findings (Eisenberg et al., 1995; Kok and Fredrickson, 2010; Geisler et al., 2013; De Witte et al., 2016). Further research should investigate the causal properties of how higher vmHRV may relate to the use of social support as an emotion regulation strategy.

One of the core theoretical and empirical underpinnings of vmHRV is that it reflects the capacity of context- and goal-based control of emotions (Thayer et al., 2012). This includes both the capacity for context-appropriate emotional responses (i.e., having a flexible autonomic nervous system), and for flexible adjustment and recovery after experiencing emotionally significant events. In RSCA, the REA is thought to capture the intensity, impairment of daily functioning, and the recovery of emotional reactivity (Prince-Embury, 2014), and is not directly targeting emotion regulation. As indicated by our hypothesis, we expected that higher vmHRV would be related to lower emotional reactivity indicated by REA, but this was not the case in the present findings. However, participants with higher vmHRV showed a tendency to have fewer problems with recovery after experiencing stress, although this was not significant. Previously studies have suggested a link between greater vmHRV and a disposition to recover more efficiently from stress (Souza et al., 2007, 2013). The small sample in the current study may explain the insignificant findings, and future work should investigate this link in more detail. Additionally, with regard to the suggestion that physiological/emotional reactivity and regulation may be independent processes (Obradović, 2012), also reflected by different physiological processes (Laborde et al., 2018), the current finding was not unexpected. The results should therefore be interpreted in light of this, and maybe explain why resting vmHRV did not correlate with REA. It has also been suggested that it is the interplay between reactivity and regulation that may predict an individual’s ability to adapt to different stressors (Obradović, 2012). For example, one study found that it was the interplay between resting vmHRV and vmHRV reactivity that could predict development of internalizing or externalizing problems 2 years after being exposed to an interpersonal stressor (Hinnant and El-Sheikh, 2009). Therefore, we argue that future work investigating the role of emotional reactivity and regulation in personal resiliency should look at both resting vmHRV and vmHRV reactivity. This is also in line with the vagal tank theory that suggests three levels of adaptability (i.e., resting, reactivity, and recovery) of cardiac vagal control (i.e., HRV) on self-regulation (Laborde et al., 2018). Given that personal resiliency is a developing process that is influenced by different biopsychosocial factors at different levels (i.e., biology, individual, family, social/community), it is more than likely that only a subset of this process will be captured by measuring resting vmHRV.

### Limitations and Future Directions

The present findings should be interpreted with regard to several limitations. The sample being investigated consisted of healthy high-school students who had chosen psychology as a subject. This together with a restricted age group and small a sample size limits the generalizability of the findings. Age, weight, and excessive cardiovascular training, may also influence vmHRV data (Leicht et al., 2003; Eyre et al., 2014). We also did not systematically control respiratory frequency, which may have influenced HRV data (Laborde et al., 2017). To address these challenges, future studies should use stratified sampling with participants from different educational directions and varied age groups, together with controlling for weight, cardiovascular training, and respiratory frequency during assessment. In addition, although gender differences in vmHRV were not present in this study, other studies indicate that this may be the case (Silvetti et al., 2001). Limited number of males may be one possible explanation and is therefore a limitation that also must be taken into account when interpreting current results. It is also worth mentioning that PPG as a physiological measurement is not the first-alternative for vmHRV analysis, but it is proven to be a highly useful approach to obtain vmHRV information (Lu et al., 2008; Jeyhani et al., 2015). As a non-invasive and low-cost technique (Allen, 2007) it was easily applied at the youth’s own high school. Additionally, the artifact correction used in the study is not optimal given the uncertainty of correctly identifying heartbeats using IBI signals (Laborde et al., 2017).

## Conclusion

The present study is among the first to apply a multilevel approach that incorporates both self-reported and psychophysiological correlates in the investigation of the role of emotion regulation in personal resiliency. First, the study did confirm Prince-Embury’s *Three-Factor model of Personal Resiliency* (Prince-Embury, 2006, 2007, 2014) with the use of an adapted version of the RSCA to a Norwegian student sample. Secondly, emotion regulatory processes measured by self-reported and psychophysiological was partly related to personal resiliency. Adaptive emotion regulation was related to protective characteristics of personal resiliency, while maladaptive emotion regulation was related to the risk characteristic of personal resiliency. However, the latter was in contrast to previous research and should be investigated in more detail. With regard to psychophysiological correlates, there was not identified any association between resting vmHRV and the global scales of RSCA. An association was found between resting vmHRV and the sub scale trust in others. *Post hoc* analysis investigating resting vmHRV sub groups (i.e., high and low vmHRV) separately indicated that adolescents with higher resting vmHRV were associated with greater self-reported sense of mastery and sense of relatedness. This was not the case for adolescents with lower vmHRV. This must, however, be interpreted with great caution because of the small sample size. This study provides preliminary findings suggesting that emotion regulatory processes may be related to personal resiliency. The correlational design together with several limitations implies that no causal conclusion can be made. We do hope, however, that the study will stimulate further research on the role of emotion regulation in personal resiliency in both non-clinical and clinical samples.

## Data Availability

Requests to access the datasets can be directed to sjursaetren@gmail.com.

## Author Contributions

SSS has contributed greatly with data collection, analyses, literature review, and writing. SS has contributed with physiological measurement and analysis, and writing the section “Heart Rate Variability – A Biological Correlate of Emotion Regulation Capacity” and helped on the discussion. RL has contributed with data collection and analyses. SP-E has helped with writing, and with great contribution on the section “The Three-Factor Model of Personal Resiliency”. GM has helped with factor analysis, and contributed on the “Materials and Methods”/“Results” sections.

## Conflict of Interest Statement

The authors declare that the research was conducted in the absence of any commercial or financial relationships that could be construed as a potential conflict of interest.
